# Maternal and Perinatal Determinants of Late Hospital Discharge Among Late Preterm Infants; A 5-Year Cross-Sectional Analysis

**DOI:** 10.3389/fped.2021.685016

**Published:** 2021-06-16

**Authors:** Wasim Khasawneh, Rahaf Alyousef, Zuhour Akawi, Areen Al-Dhoon, Ahlam Odat

**Affiliations:** Department of Pediatrics and Neonatology, Faculty of Medicine, Jordan University of Science and Technology, Irbid, Jordan

**Keywords:** late preterm, length of stay, perinatal factors, readmission, Jordan

## Abstract

**Background:** Although late preterm infants (LPIs) account for the majority of preterm births, they are mistakenly labelled and treated as “near term.” Whether longer initial hospital stay improves their outcomes and lowers readmission is controversial. The aim of this study is to identify maternal and perinatal factors associated with longer hospital stay and to assess the rate of readmission.

**Methods:** The medical records of LPIs delivered at an academic center in Jordan over a 5-year period were reviewed. They were divided according to their initial hospital stay into: Early discharge group (ED, ≤ 3 days) and late discharge group (LD, > 3 days). Maternal and perinatal factors associated with > 3-day hospital stay were reported. The rate of readmission was compared between both groups.

**Results:** 2236 LPIs were included in the analysis representing 13% of total births and 81% of premature births. LD group constituted 54%. A thousand two hundred forty three (56%) required admission to NICU. Factors associated with longer hospital stay included maternal prolonged rupture of membranes (AOR 1.9, 95% C.I 1.5, 2.4, *p* 0.000), C-section delivery (AOR 2.4, 95% C.I 1.9, 3, *p* 0.001), <35-week gestation (AOR 3.8, 95% C.I 2.6, 5, *p* 0.000), small-for-gestational age (AOR 1.9, 95% C.I 1.1, 3.8, *p* 0.03), birthweight <2,500 g (AOR 1.3, 95% C.I 1.1, 1.6, *p* 0.02), NICU admission (AOR 6.3, 95% C.I 3.4, 11.5, *p* 0.000), RDS (AOR 2.3, 95% C.I 1.5, 3.6, *p* 0.005), surfactant therapy (AOR 5, 95% C.I 1.9, 13.5, *p* 0.001), use of CPAP (AOR 1.7, 95% C.I 1.2, 2.2, *p* 0.001), jaundice (AOR 11.2, 95% C.I 7.7, 16.2, *p* 0.000), and sepsis (AOR 10.3, 95% C.I 4.8, 22, *p* 0.000). Readmission rate was 19% among the LD group and 13% among the ED group.

**Conclusion:** LPIs are at high risk for developing prematurity-related morbidities and the duration of their initial hospital stay can be anticipated based on certain predisposing maternal and perinatal factors. Late discharge of LPIs does not lower the rate of readmission.

## Introduction

Prematurity remains the leading cause of morbidity and mortality during the neonatal period and early childhood (1). The overall rate of prematurity varies between countries with rates reported between 7 and 20%. Late preterm infants (LPIs) delivered at gestational age (GA) of 34 to 36 6/7 weeks represent the most mature and the fast-growing category of prematurity (1, 2).

The rate of late preterm (LP) delivery has substantially increased over the past decade. Several reports have shown that LPIs constituted about 7–12% of all births and nearly 75% of all premature infants (3). The majority of LPIs are delivered to mothers who present with preterm labor or with premature rupture of membranes (4). Still, some LPIs are delivered due to other obstetric medical indications related to the mother or the fetus including antepartum hemorrhage, hypertension, gestational diabetes mellitus, multiple gestations, and intrauterine growth restriction (5). The increasing rates of assisted pregnancy, labor induction, and elective Cesarean section (CS) delivery have contributed to the increase in the rate of LPI delivery as well (4, 6).

Although LPIs are sometimes called near term infants, this is a misconception as this group of premature babies poses a much higher risk than term babies due to structural and functional immaturity (7). Studies have clearly documented that LPIs are at higher risk of respiratory morbidities, neonatal jaundice, feeding difficulties, hypoglycemia, temperature instability, and sepsis (8, 9). Similarly, the mortality rate and long-term neurodevelopmental outcomes are worse than term infants (10).

The outcome of LPIs has been extensively studied and reported in literature. Several reports have studied the maternal and fetal risk factors associated LPI delivery. Other reports focused on the perinatal outcomes of LPIs according to indication for delivery (5, 11).

Therapeutic and preventive interventions have been targeted toward preterm babies delivered before 34 weeks. This factor together with the falsified assumption that LPIs act like small term infants led to an underestimate of these babies' needs (7). In most practices, LPIs are admitted after delivery to the postpartum mother's room or to the well-baby nursery where they receive routine newborn care. Furthermore, in resource-limited countries like Jordan, most women tend to request discharge from the hospital within 12 h following vaginal birth and before 36 h following C-section, and they request to have their newborn babies discharged with them unless they are getting extra support in the neonatal ICU.

Several studies concluded that shorter postnatal stay is not associated with an increased rate of readmission if proper outpatient follow-up is provided (12, 13). On the other hand, short-term morbidities, readmission rates, and neonatal mortality have been linked to shorter initial hospital stay of newborns in several other reports (14). In the US, a state legislation was implemented in 1996 mandating an in-hospital observation of all newborns for a minimum of 48 h after normal vaginal delivery (15).

Besides the exact gestational age, multiple perinatal and neonatal variables are expected to affect the LOS among LPIs. Most of the reports that studied the LOS have focused on the effect of LOS on the readmission rate (16, 17). While analyzing such a potential association, it should be emphasized that infants with longer LOS often have serious health conditions that warrant higher readmission (12).

In Jordan and nearby countries, data is very scarce about LPIs care practice and outcomes. We, therefore, conducted this review to assess the maternal and perinatal factors associated with > 3-day initial LOS among LPIs who are delivered at King Abdullah University Hospital (KAUH) in North Jordan in the period 2015–2020 and to determine the effect of the initial LOS on readmission.

## Materials and Methods

We conducted a retrospective single-center analysis of all LPIs delivered at KAUH in the period 2015–2020. KAUH is the only tertiary academic center in North of Jordan that provides healthcare access for more than two million Jordanian population with an annual number of deliveries around 3,500.

Our local hospital protocol is to keep all healthy term infants and LPIs at the hospital for a minimum of 24 h after vaginal birth and 48 h after CS unless parents request earlier discharge and sign a form against medical advice. LPIs born at a GA of 34 to 34 6/7 weeks or with a birth weight of <2,000 g are initially admitted to the NICU for 48 h. Discharge criteria include stable vital signs for 24 h, stable respiratory status without requiring any oxygen support, and reasonably tolerating enteral feeding. Close follow up of serum bilirubin within 24–48 h is scheduled for all LPIs and an appointment at the outpatient clinic is scheduled within 5–7 days.

All LPIs who were delivered during the study period were included in the data collection. A list of the included infants was obtained from the hospital electronic database. Excluded from the analysis were LPIs with major congenital anomalies, chromosomal abnormalities, or with <1,500-g birthweight as these conditions are expected to require longer NICU stay and might contribute to skewness of the results.

Data collection was conducted by well-trained interns and medical students using pretested variables and documented in an excel spreadsheet.

Collected maternal variables include age, parity, employment, previous CS delivery, assisted pregnancy, antenatal care attendance, antenatal steroid, and pregnancy complications. Delivery related variables include gestational age, induction of labor, prolonged premature rupture of membranes (PPROM), mode of delivery, and type of anesthesia.

Neonatal variables include gender, birth weight, weight for GA status, and whether a singleton or multiple gestation.

We also collected data about neonatal outcomes including Apgar score, admission to NICU, morbidities like respiratory morbidity and support, hypoglycemia, hypothermia, neonatal jaundice, sepsis, congenital anomalies, discharge weight, duration of hospitalization, and readmission within 28 days after discharge.

Respiratory distress syndrome (RDS) and transient tachypnea of the newborn (TTN) were diagnosed in babies with signs of respiratory distress and typical CXR findings (18). Neonatal jaundice was diagnosed and treated according to the AAP guidelines (19). The term small-for-gestational age (SGA) was used for any baby with a birth weight below the 3^rd^ centile for age based on Fenton growth chart, while large-for-gestational age (LGA) was used if it falls above the 97^th^ centile (20).

LPIs were divided into two groups, the “early discharge” group (ED) refers to those who were discharged from the hospital within 3 days from the time of birth. The “late discharge” group (LD) refers to those who had an initial LOS of more than 3 days. We chose 3 days as the cutoff since the median length of stay among our cohort was 3 days. Both groups were compared in terms of maternal and perinatal characteristics and neonatal outcomes including readmission rate.

The primary outcome was to assess maternal and perinatal factors associated with longer hospital stay or late discharge beyond 3 days. The secondary outcomes were the rates of morbidities such as RDS, respiratory support, hypothermia, hypoglycemia, jaundice, sepsis, and readmission rate.

Statistical analysis was performed using IBM SPSS Statistics Version 25 (Armonk, NY, IBM Corp). Data were presented as frequency distributions and percentages for categorical variables and mean ± standard deviation (SD) for continuous variables. Pearson χ2 test was used to evaluate the significance of association between categorical variables, while student's *t*-test and one-way ANOVA were applied to examine the significance level for continuous normally distributed variables. A *P*-value ≤ 0.05 was considered statically significant. If a significant relationship was observed, a *post-hoc* residual analysis for categorical variables and a Fisher's test for continuous variables were applied to determine the exact significance between groups for each variable. After identification of maternal, neonatal, and perinatal factors with significant association with a LOS of > 3 days based on *p*-values of <0.05, a binary logistic regression model was performed to study the multiple effects of different variables on the LOS. Adjusted OR with 95% C.I were accordingly reported.

## Results

A total of 2,265 LPIs were delivered at KAUH during the 5-year study period representing a rate of 13% of the total number of births (17,420) and 81% of the total premature births (2,796). Twenty-nine LPIs were excluded (four with chromosomal anomalies, five with neural tube defects, one with congenital diaphragmatic hernia, two with intestinal atresia, two with complex congenital heart disease, and 15 VLBW <1,500 g). Of the 2,236 LPIs included in analysis, 1,137 (51%) were males and 1,915 (86%) were born after 35-week gestation. The majority (88%) of LPIs were appropriate-for-gestational age with a mean birth weight of 2,575 (± 485) g while only 185 (8.3%) were SGA.

Mothers of the included LPIs were mostly 21–35 years old (1,642, 73%), multiparous (1,715, 76%), and non-employed (1792, 80%). Antenatal care attendance was ascertained in 989 (44%) and antenatal steroids were administered for 740 (33%). Five hundred thirty-five (24%) presented with PPROM, 282 (13%) required induction of labor for medical reasons while 1,719 (77%) delivered by Cesarean section (CS) of whom 716 had an emergency procedure (Table 1).

**Table 1:** Maternal and neonatal characteristics.

**Maternal**		***N* = 2,236**	**%**
Age (years)	≤ 20	52	2.3
	21–35	1,642	73.4
	>35	542	24.2
Parity	Primiparous	521	23.3
	Multiparous	1,715	76.7
Employment		444	20
Assisted pregnancy		322	14.4
Adequate antenatal care		989	44.2
Previous Cesarean section		1,010	45.2
Antenatal steroids		740	33
Pregnancy complications	None	1,654	74
	Pre-eclampsia	127	5.7
	Gestational DM	93	4.2
	Placenta Previa	95	4.2
	Placental abruption	20	1
	Chorioamnionitis	12	0.5
	Others	235	10.5
Induction of labor	Yes	282	13
PPROM	Yes	535	24
Mode	Vaginal	517	23
	Elective CS	1,003	45
	Emergency CS	716	32
Anesthesia	None	517	23
	General	507	23
	Spinal/epidural	1,212	54
**Neonatal**			
Gender	Male	1,137	51
	Female	1,099	49
Gestational age	<35	321	14
(weeks)	≥35	1,915	86
	34	321	14
	35	551	25
	36	1,364	61
Birth weight	≤ 2,500	1,052	47
(grams)	>2,500	1,184	53
Weight/gestational age	AGA	1,975	88.3
	SGA	185	8.3
	LGA	76	3.4
Multiple gestation	yes	522	23

*DM, diabetes mellitus; CS, cesarean section; PPROM, prolonged premature rupture of membranes; AGA, appropriate for gestational age; SGA, small for gestational age; LGA, large for gestational age*.

More than half (54%, 1,216) stayed at the hospital for > 3 days (LD group) and 1,243 (56%) required NICU admission. Compared with the ED group, LPIs in the LD group were more likely to have a GA of <35 weeks (22 vs. 6%, *p* < 0.005), a birth weight below 2,500 g (54 vs. 39%, *p* < 0.005), be SGA (11 vs. 5%, *p* < 0.005), and be a product of multiple gestation pregnancy (26 vs. 20%, *p* = 0.001). Maternal and perinatal factors of significant association with LD included non-employment (83 vs. 77%, *p* = 0.001), assisted pregnancy (18 vs. 10%, *p* < 0.005), antenatal steroids (39 vs. 26%, *p* < 0.005), pregnancy complications (30 vs. 22%, *p* < 0.005), maternal PPROM (28 vs. 19%, *p* < 0.005), CS delivery (83 vs. 70%, *p* < 0.005), and the use of general anesthesia (25 vs. 20%, *p* < 0.005) (Table 2).

**Table 2:** A comparison of maternal and perinatal factors according to the length of stay.

**Maternal**		**ED group *N* = 1,020**	**LD group hours *N* = 1,216**	***P*-value**
		***N* (%)**	***N* (%)**	
Age (years)	≤ 20	28 (3)	24 (2)	ns
	21–35	746 (73)	896 (74)	
	>35	246 (24)	295 (24)	
Parity	Primiparous	219 (21)	302 (25)	ns
	Multiparous	801 (79)	914 (75)	
Employment		235 (23)	209 (17)	0.001
Assisted pregnancy		107 (10)	215 (18)	0.000
Adequate antenatal care		467 (46)	522 (43)	ns
Previous Cesarean section		438 (43)	572 (47)	ns
Antenatal steroids		265 (26)	475 (39)	0.000
Pregnancy complications	None	797 (78)	857 (70)	0.000
	Pre-eclampsia	46 (5)	81 (7)	
	Gestational DM	30 (3)	63 (5)	
	Placenta Previa	30 (3)	65 (5)	
	Placental abruption	6 (<1)	14 (1)	
	Chorioamnionitis	5 (<1)	7 (<1)	
	Others	106 (10)	129 (11)	
Induction of labor		139 (14)	143 (12)	ns
PPROM		197 (19)	338 (28)	0.000
Mode of delivery	Vaginal	312 (30)	205 (17)	0.000
	Elective CS	415 (41)	588 (48)	
	Emergency CS	293 (29)	423 (35)	
Anesthesia	None	314 (31)	203 (17)	0.000
	General	201 (20)	306 (25)	
	Spinal/epidural	507 (49)	707 (58)	
**Neonatal**				
Gender	Male	520 (51)	617 (51)	ns
	Female	500 (49)	599 (49)	
Gestational age	<35	59 (6)	262 (22)	0.000
(weeks)	≥35	961 (94)	954 (78)	
	34	59 (6)	262 (22)	0.000
	35	228 (22)	323 (26)	
	36	733 (72)	631 (52)	
Birth weight	≤ 2,500	398 (39)	654 (54)	0.000
	>2,500	622 (61)	562 (46)	
Weight/gestational age	AGA	939 (92)	1036 (86)	0.000
	SGA	46 (5)	139 (11)	
	LGA	35 (3)	41 (3)	
Multiple gestation		205 (29)	317 (26)	0.001

*ED, early discharge; LD, late discharge; DM, diabetes mellitus; CS, cesarean section; PPROM, prolonged premature rupture of membranes; AGA, appropriate for gestational age; SGA, small for gestational age; LGA, large for gestational age*.

Table 3 shows the clinical outcomes of the included infants. One hundred and six (5%) had a 5-min Apgar score of <7, 509 (23%) were diagnosed with RDS or TTN, 137 (7%) received surfactant, 373 (17%) developed jaundice requiring therapy, 102 (5%) had sepsis, 40 (22%) had hypoglycemia, and only 10 (<1%) had hypothermia. Of the 362 (16%) LPIs who were readmitted to the hospital within 28 days after discharge, 230 belong to the LD group accounting for a rate of 19% compared with 132 (13%) among the ED group (*p*-value 0.001). The main indications for readmission were neonatal jaundice and suspected sepsis. The median age at readmission was 10 (25–75% IQR 5, 16).

**Table 3:** Neonatal outcomes of late preterm infants.

**Outcome**		***N* = 2,236**	**ED group *N* = 1,020**	**LD group hours *N* = 1,216**	***P*-value**
		***N* (%)**	***N* (%)**	***N* (%)**	
APGAR	<7	106 (5)	22 (2)	84 (7)	0.000
	≥7	2,130 (95)	998 (98)	1,132 (93)	
NICU admission		1243 (56)	297 (29)	946 (78)	0.000
RDS		390 (17)	45 (4)	345 (28)	0.000
TTN		119 (5)	26 (2)	93 (8)	0.000
Surfactant therapy	1 dose	80 (4)	6 (<1)	74 (6)	0.000
	≥ 2 doses	57 (3)	0 (0)	57 (5)	
Invasive ventilation		9 (<1)	5 (<1)	4 (<1)	
CPAP		700 (31)	119 (12)	581 (48)	0.000
Hypoglycemia		40 (2)	12 (1)	28 (2)	0.04
Hypothermia		10 (<1)	3 (<1)	7 (<1)	
Jaundice		373 (17)	33 (3)	340 (28)	0.000
Sepsis	Presumed	83 (4)	7 (<1)	76 (6)	0.000
	Confirmed	19 (1)	0 (0)	19 (2)	
Readmission		362 (16)	132 (13)	230 (19)	0.000

*ED, early discharge; LD, late discharge; NICU, neonatal intensive care unit; RDS, respiratory distress syndrome; TTN, transient tachypnea of newborn; CPAP, continuous positive airway pressure*.

After identification of maternal, neonatal, and perinatal factors with significant association with a LOS of > 3 days based on *p*-values of <0.05, a binary logistic regression model was performed and the factors with persistent significance were reported. Determinants of a > 3-day LOS included maternal PPROM (AOR 1.9, 95% C.I 1.5, 2.4, *p* 0.000), CS delivery (AOR 2.4, 95% C.I 1.9, 3, *p* 0.001), GA <35 weeks (AOR 3.8, 95% C.I 2.6, 5, *p* 0.000), SGA (AOR 1.9, 95% C.I 1.1, 3.8, *p* 0.03), birth weight < 2,500 g (AOR 1.3, 95% C.I 1.1, 1.6, *p* 0.02), NICU admission (AOR 6.3, 95% C.I 3.4, 11.5, *p* 0.000), RDS (AOR 2.3, 95% C.I 1.5, 3.6, *p* 0.005), surfactant therapy (AOR 5, 95% C.I 1.9, 13.5, *p* 0.001), use of CPAP (AOR 1.7, 95% C.I 1.2, 2.2, *p* 0.001), jaundice (AOR 11.2, 95% C.I 7.7, 16.2, *p* 0.000), and sepsis (AOR 10.3, 95% C.I 4.8, 22, *p* 0.000) (Table 4).

**Table 4:** Binary logistic regression of maternal and perinatal factors associated with late discharge > 72 hours among late preterm infants.

**Factor**		**AOR**	**95% C.I**.	***P*-value**
Maternal PPROM	Yes	1.9	1.5, 2.4	0.000
	No	Ref		
Mode of delivery	C-section	2.4	1.9, 3	0.001
	Vaginal	Ref		
Gestational age	<35	3.8	2.6, 5	0.000
	≥35	Ref		
Weight/gestational age	SGA	1.9	1.1, 3.8	0.03
	AGA	Ref		
Birth weight	<2500	1.3	1.1, 1.6	0.02
	≥2500	Ref		
NICU admission	Yes	6.3	3.4, 11.5	0.000
	No	Ref		
RDS	Yes	2.3	1.5, 3.6	0.005
	No	Ref		
Surfactant therapy	yes	5	1.9, 13.5	0.001
	No	Ref		
CPAP	Yes	1.7	1.2, 2.2	0.001
	No	Ref		
Jaundice	Yes	11.2	7.7, 16.2	0.000
	No	Ref		
Sepsis	Yes	10.3	4.8, 22	0.000
	No	Ref		

*AOR, adjusted odds ratio; PPROM, prolonged premature rupture of membranes; AGA, appropriate for gestational age; SGA, small for gestational age; NICU, neonatal intensive care unit; RDS, respiratory distress syndrome; CPAP, continuous positive airway pressure*.

## Discussion

In this single-center 5-year retrospective analysis from a major tertiary center in Jordan, we reported that LPIs accounted for 13% of the total births and 81% of the premature births. This study analyzed the maternal and perinatal factors associated with late hospital discharge > 3 days and its effect on readmission. We identified maternal PROM, C-section delivery, <35-week gestation, SGA, birthweight <2,500 g, NICU admission, RDS, surfactant therapy, use of CPAP, jaundice, and sepsis as major factors associated with late discharge and concluded that a longer initial hospital stay does not necessarily lower the incidence of readmission.

Globally, the rate of prematurity has not been significantly decreased despite the advance in neonatal care. However, there has been a shift in the level of prematurity with a substantial rise in the number of LP births over the past 5–10 years (1), (2). In 2019, the US rate of LP birth was reported to be about 7.5% among all births and 73% among premature births (2, 3). In Saudi Arabia, Al-Qurashi et al. reported a prematurity rate of 6.5% with 75% being LPIs (21). In Jordan, the rate of LP birth was previously reported in 2010 from a single-center review over one-year period and found to be 7.8% (22). The higher rate of 13% reported in our analysis could be attributed to the fact that our center is the only tertiary center in North Jordan where most women with high-risk pregnancies including preterm labor, PPROM, and multiple gestations are transferred for delivery.

Our study showed that LPIs delivered before 35-week gestation or with low birth weight (LBW; <2,500 g) particularly small for gestational age (SGA) infants were more likely to stay at the hospital for more than 3 days. These factors remained significant after correction for confounders in binary logistic regression model. Factors affecting the LOS have been previously studied, but most studies focused mainly on comparing LPIs with very preterm infants or LPIs with early term infants and concluded that LOS increases by decreasing GA (12, 23). In a national US study about factors affecting LOS among LPIs, Aly et al. reported that <35-week gestation and LBW were significantly associated with a longer LOS (24). The prediction of longer LOS among this high-risk group of premature infants based on certain clinical and demographic factors might be utilized as a core element to establish healthcare guidelines that focus on provision of high-quality care, modification of discharge criteria, and improving the antenatal counseling of pregnant mothers with potential delivery before term gestation considering their cultural background and preferences.

Our study highlighted that maternal PPROM and NICU admission were associated with late discharge of LPIs. Although PPROM is an indication for NICU admission in our hospital protocol, it should be clarified that our local policy is to admit all infants delivered after PPROM to the NICU for observation immediately after delivery and to be discharged after 48 h if they meet the discharge criteria. The overall rate of NICU admission among the LPIs included in our cohort was 56% which is quite similar to the rate reported from USA (56%)(25) and from Egypt (53%) (26), a nearby Arab country, compared with 30% in Turkey (27). In a previous report, we found that maternal PPROM was the major indication of NICU admission among term infants (28).

The rate of Cesarean section delivery in the current study was 77%. This very high rate can be explained by the high percentage of high-risk pregnant women who are referred to our center for delivery including women with a previous CS (1,010, 45.2%). We found CS delivery to be a major determinant of longer LOS. This finding is in line with what has been previously reported in many studies (24, 29). For example, in the USA, Aly et al. reported that CS was associated with longer LOS among LPIs regardless of their GA category. Besides the increase in maternal request, many obstetric care providers tend to perform early CS in high-risk pregnancies mainly in multiple gestations and after assisted reproduction (30). The compliance with the American College of Obstetrics and Gynecologists' (ACOG) recommendation regarding avoiding elective CS delivery at late preterm and early term infants should be always emphasized in obstetric centers to avoid preventable prematurity-related morbidities among this high-risk group of infants in order to decrease their LOS and improve their outcomes (31).

The present study highlighted the main morbidities identified in this high-risk group. RDS, TTN, hyperbilirubinemia, and sepsis were the main complications encountered during NICU stay. Most LPIs who developed these complications stayed at the NICU beyond 3 days. This finding is not different from the mounting reports published previously from all over the world which indicated that LPIs have more complications than term infants (5, 32, 33). The rates of hypoglycemia (1.8%) and hypothermia (<1%) among our cohort were much lower than other studies (7, 8). This emphasizes the importance of local hospital policies and protocols of handling of LPIs, implementing skin-to-skin care with the mother, and early initiation of breastfeeding and frequent enteral feedings in stable infants. In the US, Baker compared the LPIs' outcomes between 2008 and 2013 at an urban Medical Center and concluded that the implementation of clinical practice guidelines in caring for LPIs had reduced the overall LOS and decreased the rates of hypoglycemia and hypothermia (34).

The high risk of readmission among LPIs has been well studied and potentially explained by the underestimate of their needs in the immediate postnatal period (14, 16, 17). Evidence about safe early newborn discharge is inconclusive. In infants who meet the criteria for discharge, it is not clear if longer length of stay (LOS) to provide longer observation of the newborns and maternal counseling would improve their outcomes. In our cohort, the mean and median initial LOS were 4.6 (±6) and 3 (IQR 2,5) days respectively with a readmission rate of 16%. In a smaller review from Lebanon, the rate of readmission was 7.9% following early discharge before 48 h (35). The main indications were consistent with most other reports worldwide and included hyperbilirubinemia and sepsis evaluation due to feeding difficulties (14), (36). Several studies addressed the rate of readmission among LPIs and considered it as a main indicator about the quality of care provided to this high-risk group. Escobar et al. reported a three-fold increase in the rate of readmission among LPIs compared with term infants and concluded that infants who were not admitted to the NICU or those with short NICU stay were more likely to be readmitted within 2 weeks This was more obvious in the more mature 36-week infants (17). Despite that, there is a conflicting evidence about the effect of initial LOS on readmission rates (37). Although longer initial hospitalization might theoretically prevent readmission, discharge from the hospital should not be postponed for infants who meet the discharge criteria. In the present study, the rate of readmission was 19% among the “LD” group compared with 13% among the “ED” group. This could be explained by the fact that LPIs who stayed longer were sicker and so posed a higher risk of having more serious complications. Same finding was highlighted in a national study from UK about all neonatal admissions at National Health Service hospitals reporting a rate of 10.6% among LPIs with a higher risk reported among those with a longer initial LOS (37). The risk of rehospitalization among LPIs extends beyond the neonatal period (38). McLaurin et al. studied the rate of rehospitalization in the first year of life and reported a rate of 15.2% with an increased chance of readmission in neonates with a >4-day initial hospital stay (39). Since most LPIs' readmissions reported by different studies are due to worsening jaundice and suspected sepsis with poor feeding, it is vital to council mothers about the proper breastfeeding technique and frequency of feeding and to arrange for close follow up at the outpatient clinic after discharge (40).

Although the number of LPIs included in our analysis exceeds the number included in many other reports, this study is not without limitations. The main limitation is being a single-center retrospective review where the accuracy of the documented data could not be validated in all cases. The poor generalizability of our results is another limitation for the same reason. Stratification of LPIs according to different gestational ages and reporting the LOS determinants, clinical morbidities, and readmission rates accordingly might give a more valid insight to their outcomes given the innate difference in physiology and functional maturity encountered with each week change in gestational age.

In conclusion, late preterm birth constitutes the main category of prematurity and LPIs remain at risk for prematurity-related morbidities and subsequent readmission. Although certain maternal and perinatal factors can be utilized to anticipate the length of initial hospitalization, a longer initial hospital stay does not necessarily lower the incidence of readmission.

### Implications and Suggestions

Local hospital protocols should be adjusted to deliver an ongoing assessment of LPIs to identify their inherent risks in order to provide them with appropriate care that meets their actual needs. Implementing policies to avoid elective deliveries at late preterm and early term gestation is essential. Quality improvement initiatives addressing the discharge timing and close outpatient follow up are key points to improve the overall outcome and to minimize the readmission rates. A multi-center future research is needed to better understand the outcome of LPIs at a national level.

## Data Availability Statement

All data collected is available from the corresponding author upon reasonable request.

## Ethics Statement

The studies involving human participants were reviewed and approved by The Institutional Review Board at Jordan University of Science and Technology. Written informed consent for participation was not provided by the participants' legal guardians/next of kin because: this work involves chart review and no direct contact with patients. Deidentified data were used all through.

## Author Contributions

WK made substantial contributions to conception and design, analysis and interpretation of data, and involved in drafting the manuscript or revising it critically for important intellectual content. RA, ZA, AA-D, and AO made substantial contribution to the acquisition and interpretation of data and were involved in drafting the manuscript. All authors gave final approval of the version to be published and agreed to be accountable for all aspects of the work.

## Conflict of Interest

The authors declare that the research was conducted in the absence of any commercial or financial relationships that could be construed as a potential conflict of interest.
